# Supplementary material for: Lipid Profiles from Dried Blood Spots Reveal Lipidomic Signatures of Newborns Undergoing Mild Therapeutic Hypothermia after Hypoxic-Ischemic Encephalopathy
Source: Nutrients. 2021 Nov 28;13(12):4301. doi: 10.3390/nu13124301 (PMC8703828; doi:10.3390/nu13124301)
Supplement: Supplementary file 1 [file nutrients-13-04301-s001.zip › nutrients-1407762-supplementary.pdf]

Supplementary Material

Table S1 This table summarises all eight lipid classes and their functions (60) and a diagram of lipid metabolism pathways (61). Within the eight lipid classes, there are lipid species. These are lipids with various structural combinations (e.g. location of double bonds or attachment of groups) based around the key structural features that define the class (e.g. the head group or the backbone). The notation for the lipid species is the abbreviation for the lipid class followed by (C-atoms : double bonds), for example TG (34:1). Abbreviations: polyunsaturated fatty acids (PUFAs), lysophospatidylethanolamine (LPE), DHA – (phosphatidylethanolamine (PE), phosphatidylserine (PS), diglyceride (DG), phosphatidic acid (PA), sphingomyelin (SM), and ceramide (Cer).

Lipid Class	Example lipid species	Function
**Fatty acyls [FA]**	PUFAs produce prostaglandins, leukotrienes and thromboxane A2.	Docosahexaenoic acid (DHA) is crucial for brain structure and function. Prostaglandins are involved in vasodilation, inflammation and pain. Thromboxane A2 is important in platelet aggregation.
**Glycerolipids [GL]**	Monoglycerol (MG), Diglyceride (DG) and Triglyceride (TG)	They are the main form of energy store, especially as fat in mammals. DG is a key signalling molecule, as it is able to diffuse easily in and out of the cell.
**Glycerophospholipids [GP]**	Phosphatidylcholine (PC), Phosphatidylethanolamine (PE), Phosphatidylinositol (PI) and Plasmalogen	Phospholipids are the main component of the cell membrane and help make them permeable to allow for flow of chemical messengers and nutrients into and out of the cell. PC is the most abundant phospholipid in cells, especially within cell membranes. Glycerophospholipids have a glycerol head group.
**Sphingolipids [SP]**	Ceramide (Cer) and Sphingomyelin (SM)	Cer can form of sphingomyelin, cerebrosides and gangliosides. SM is found in myelin sheaths. Sphingolipids are important in transmembrane signaling, differentiation, apoptosis and cell survival.
**Saccharolipids [SL]**	Glucosamine	Interact with the cell membrane.
**Polyketides [PK]**	Doxycycline and erythromycin	They are secondary metabolites. Many have immunosuppressant or antimicrobial properties.
**Sterol lipids [ST]**	Cholesterol (Chol), steroids and bile acids	Chol is embedded in cell membranes and is important in structural integrity and fluidity.
**Prenol lipids [PR]**	Carotenoids and Quinones	They form fat soluble vitamins - A, D, E and K.

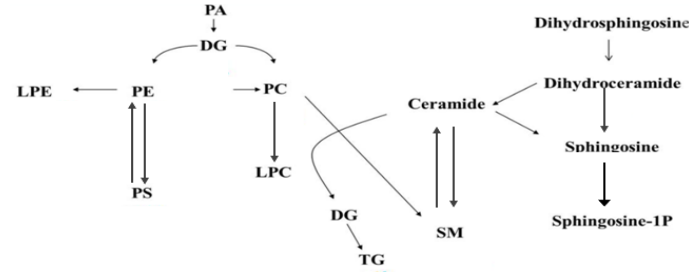

Table S2 Nutrition groups of the studied population at S1, S2 and S3

Type of nutritional feed	Mild HIE No TH	Moderate-Severe HIE TH		
	**S1**	**S1**	**S2**	**S3**
**Intravenous Dextrose**	7	35	17	9
**Breast Milk**	5	0	18	21
**Formula**	3	2	4	4
**Mixed (BM + formula)**	3	0	4	6
**Parenteral nutrition**	0	2	12	13
